# The effect of concentric constriction of the visual field to 10 and 15 degrees on simulated motor vehicle accidents

**DOI:** 10.1371/journal.pone.0193767

**Published:** 2018-03-14

**Authors:** Sachiko Udagawa, Shinji Ohkubo, Aiko Iwase, Yuto Susuki, Shiho Kunimatsu-Sanuki, Takeo Fukuchi, Chota Matsumoto, Yuko Ohno, Hiroshi Ono, Kazuhisa Sugiyama, Makoto Araie

**Affiliations:** 1 Department of Ophthalmology and Visual Science, Kanazawa University Graduate School of Medical Science, Ishikawa, Japan; 2 Tajimi Iwase Eye Clinic, Gifu, Japan; 3 Department of Robotics & Design for Innovative Healthcare Graduate School of Medicine, Osaka University, Osaka, Japan; 4 Department of Ophthalmology, Tohoku University Graduate School of Medicine, Miyagi, Japan; 5 Department of Ophthalmology, Niigata University, Niigata, Japan; 6 Department of Ophthalmology Faculty of Medicine Kindai University, Osaka, Japan; 7 Honda Motor Co., Tokyo, Japan; 8 Kanto Central Hospital of the Mutual Aid Association of Public School Teachers, Tokyo, Japan; Tokai University, JAPAN

## Abstract

**Purpose:**

Traffic accidents are associated with the visual function of drivers, as well as many other factors. Driving simulator systems have the advantage of controlling for traffic- and automobile-related conditions, and using pinhole glasses can control the degree of concentric concentration of the visual field. We evaluated the effect of concentric constriction of the visual field on automobile driving, using driving simulator tests.

**Methods:**

Subjects meeting criteria for normal eyesight were included in the study. Pinhole glasses with variable aperture sizes were adjusted to mimic the conditions of concentric visual field constrictions of 10° and 15°, using a CLOCK CHART^®^. The test contained 8 scenarios (2 oncoming right-turning cars and 6 jump-out events from the side).

**Results:**

Eighty-eight subjects were included in the study; 37 (mean age = 52.9±15.8 years) subjects were assigned to the 15° group, and 51 (mean = 48.6±15.5 years) were assigned to the 10° group. For all 8 scenarios, the number of accidents was significantly higher among pinhole wearing subjects. The average number of all types of accidents per person was significantly higher in the pinhole 10° group (4.59±1.81) than the pinhole 15° group (3.68±1.49) (P = 0.032). The number of accidents associated with jump-out scenarios, in which a vehicle approaches from the side on a straight road with a good view, was significantly higher in the pinhole 10° group than in the pinhole 15° group.

**Conclusions:**

Concentric constriction of the visual field was associated with increased number of traffic accidents. The simulation findings indicated that a visual field of 10° to 15° may be important for avoiding collisions in places where there is a straight road with a good view.

## Introduction

**Fig 1:**
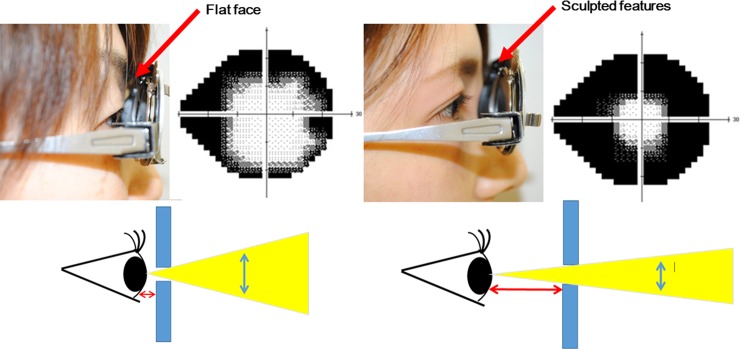
Visual field constriction by pinhole glasses. Subjects with normal eyesight wore pinhole (PH) glasses with a 2 mm aperture that caused VF constriction. Of note, PH glasses with the same aperture can lead to a different level of VF constriction between individuals.

**Fig 2:**
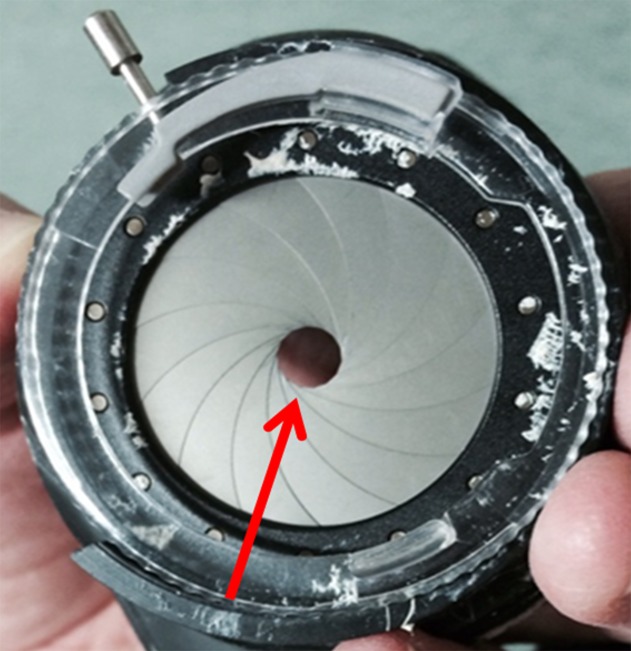
PH glasses with variable apertures. We developed PH glasses with variable apertures to create restricted visual fields.

Integrity of visual function is essential to ensuring safe driving and avoiding motor vehicle accidents (MVAs). Retinitis pigmentosa and glaucoma of advanced stage are conditions accompanied by serious visual field (VF) impairment and persons with these conditions are more likely to be involved in MVAs [1–12]. Safe driving, however, depends not only on the visual function of subjects, but also on many other factors, including traffic- and automobile-related conditions and subject-related conditions, such as individual driving skill, reflexes, or alertness. Driving simulator (DS) systems have advantages in controlling traffic- and automobile-related conditions [1,13,14]. Driving, however, is a complex activity involving sensory, motor, and cognitive functions [15]. In addition to VF integrity, many other factors, such as reaction time and driving technique, also influence an individual’s driving performance. As such, it is difficult to correct for the effects of confounding factors, other than VF damage, when comparing results obtained for subjects with VF impairment and to those of normal subjects without VF impairment. However, VF impairment can be artificially created by having subjects wear pinhole (PH) glasses. Comparison of involvement in MVAs during DS tests [3] between the same subjects with and without PH glasses would allow investigation of the effects of VF impairment, while adjusting for all traffic-, automobile- and subject-related conditions other than VF impairment. It is important to note that the PH of the same aperture can create a different level of VF constriction between individuals (Fig 1). We devised PH glasses with variable PH apertures to create specific visual angles (Fig 2).

In the current study, we simulated concentric constriction of binocular VFs to central 10° or 15° with PH glasses. We then studied the effects of concentric constriction of binocular VFs on MVAs, after adjusting for effects of other confounding factors, using a DS system for which utility in studying visual function and MVA relationship had been previously confirmed [3].

## Material and methods

### Study design

The current study was approved by the Ethical Committee of the Gifu Prefecture Medical Association (TJMIW-201301). Written informed consent was obtained from all participants. The participants in this study have also given written consent to publish the photograph show in Fig 1. All aspects of the protocol conformed to the tenets of the Declaration of Helsinki. This was conducted as one of the investigative studies of the National Police Agency, and we obtained permission to use the data and publish the results.

### Ocular examination protocol

We included 99 healthy subjects whose visual fields were confirmed to be normal with the Humphrey Visual Field Analyzer Swedish Interactive Threshold Algorithm standard 24–2 (HFA 24–2, Carl Zeiss Meditec Inc., Dublin, California). All subjects were examined at the Tajimi Iwase Eye Clinic between October 2013 and December 2013 under identical conditions. All subjects received a complete ophthalmologic examination, including a slit lamp examination, intraocular pressure (IOP) measurement using a Goldmann applanation tonometer, an ophthalmoscopy examination, best-corrected visual acuity (BCVA) assessment, and standard automated perimetry with the HFA 24–2.

### Exclusion criteria

Exclusion criteria for ocular examination were BCVA <1.0; IOP >21 mmHg, and those subjects with ocular diseases, strabismus, amblyopia, history of ocular surgery including laser therapy, and history of systemic or neurologic disease.VF exclusion criteria were fixation loss > 20%, false negative error or false positive error > 15% or abnormal VF test results, according to Anderson and Patella [16], defined as the presence of at least one of the following criteria: 1) a pattern deviation probability plot showing a cluster of 3 or more points with a probability of less than 5% and at least one point with a probability less than 1% in the cluster; 2) pattern standard deviation with a probability of less than 5%; 3) glaucoma hemifield test that indicated that the field was outside normal limits.

### Simulation of concentric constriction of binocular VF to central 15° and 10°

**Fig 3:**
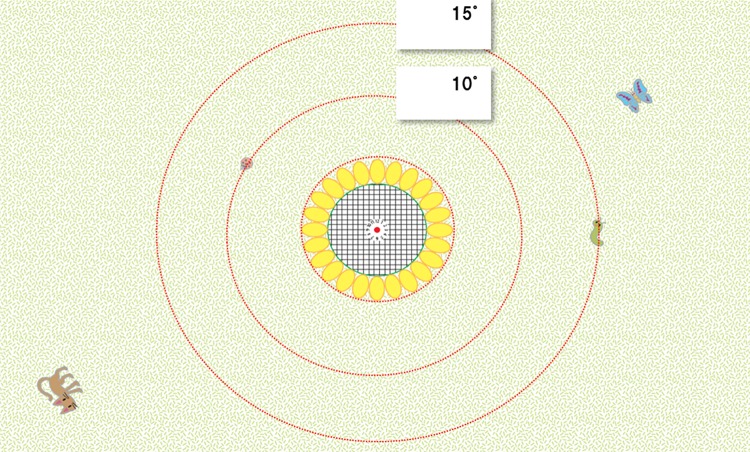
CLOCK CHART^®^. The VF constrictions of 10° and 15° were created using a pinhole with variable apertures and a CLOCK CHART^®^ displaying a ladybug and a caterpillar on the central 10°and 15°eccentricity, respectively.

PH glasses with variable apertures were made to simulate concentric constriction of binocular VF (Fig 1) as measured with a multiple-stimulus self-check VF screener (CLOCK CHART^®^)[17]. The PH apertures and inter-eye distance of PH were individually adjusted to ensure binocular vision and to ensure that only an area of central 15° or 10° on the CLOCK CHART^®^ could be seen (Fig 3). The participants were randomly allocated to the PH central 15°-VF (PH15) group or the PH central 10°-VF (PH10) group.

### Driving simulator

A DS system (HONDA Safety Navi ‘glaucoma edition 2’, Honda Motor Co., Tokyo) was used for which details have been previously reported [2–3]. The simulation used a semi-automatically-controlled speed; subjects only had to brake in case of a vehicle suddenly appearing.

Before using the DS, all participants completed a questionnaire to determine their: (1) age and sex; (2) driving history (years since acquisition of first driving license); (3) number of times driving in a month; and (4) time spent driving per day. The DS test was performed between 10:00 am and 5:00 pm. The subjects participated in a 2-minute practice session, without PH glasses, followed by a 5-minute test without PH glasses (PH-). After a 10-minute rest, the subjects completed a 5-minute test with PH glasses. All procedures were completed within 30 minutes so that the effects of circadian rhythm on driving performance [18,19] could be minimized upon comparison of the results obtained from the same subjects both with and without PH glasses were compared. If a subject reported experiencing sickness or fatigue caused by the simulator during the practice or main session, further testing was immediately abandoned. We instructed subjects to maintain binocular fixation on a point in the center of the screen, and not to move their head. In this study, subjects with PH glasses could only see the screen through the PHs. If subjects moved their gaze, they could not see through the PHs, because the inter-eye distance of the PHs was locked. The examiner checked the head movement of the subject, and alerted subjects not to move their heads during test time.

**Fig 4:**
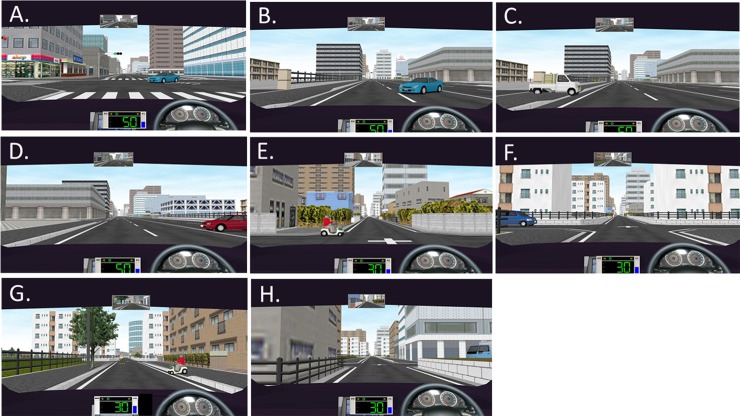
Screenshots of simulated scenarios. The main test contained 8 scenarios depicting situations such as oncoming right-turning cars (A, B) and suddenly appearing hazards from the side in front of the car (C-H) Screenshots of the 8 scenarios: A. Oncoming right-turning blue car 1. B. Oncoming right-turning blue car 2. C. White car appearing from left. D. Red car appearing from right. E. Mobility scooter appearing from left. F. Blue car appearing from left. G. Mobility scooter appearing from right. H. Blue car appearing from right.

The main test contained 8 scenarios depicting situations such as oncoming right-turning cars (2 scenarios) and suddenly appearing hazards from the side in front of the car (6 scenarios) (Fig 4). We recorded the number of collisions that took place during each of the 8 scenarios.

### Statistical analysis

The mean number of accidents and standard deviations were calculated and compared between the PH15 and PH- normal conditions and between the PH10° and PH- normal conditions. The number of accidents in the PH15 and PH10 groups was also compared. Kolmogorov-Smirnov tests were used to evaluate the distribution of numerical data for parametric characteristics. As most of the parameters were not found to be normally distributed, comparison between groups was carried out using non-parametric methods. Fisher's exact test was used to analyze the number of accidents in each scenario, and within each group, by comparing results obtained with PH glasses to those obtained without PH glasses. Statistical analysis was performed with SPSS software version 22.0 (SPSS Inc., Chicago, Illinois, USA). P values less than 0.05 were considered statistically significant.

## Results

Of the 99 subjects, 2 could not complete the practice session due to simulator sickness and were excluded; 9 additional subjects who completed the practice session were excluded when they experienced simulator sickness during the main test. No participants reported fatigue during the main test. Therefore, 88 subjects (41 male and 47 female) completed the main DS tests. Comparison between the PH15 and PH- normal conditions was performed for 37 subjects, while comparison between the PH10 and PH- normal conditions was performed for 51 subjects. There was no significant difference in age, gender, BCVA, spherical equivalent, driving history, number of times driving per month, or driving time per day between the PH10 and PH15 groups (Table 1).

**Table 1:** Subject characteristics.

	PH10 group (51 cases)	PH15 group (37 cases)	P value
Demographic			
Age (years), mean ± SD (range)	48.6 ± 15.5	52.9 ± 15.8	0.344^‡^
	(23–75)	(23–78)	
Gender (female/ male)	23 / 28	14 / 23	0.086^†^
Right eye spherical equivalent refractive error (diopters), mean ± SD (range)	-2.5 ± 3.3	-2.2 ± 1.3	0.916^‡^
	(-15.0 - +1.5)	(-7.8 - +1.9)	
Left eye spherical equivalent refractive error (diopters), mean ± SD (range)	-2.3 ± 3.3	-1.9 ± 2.6	0.829^‡^
	(-15.0 - +1.5)	(-7.8 - +2.3)	
Driving characteristics			
Driving history (years), mean ± SD (range)	28.8 ± 15.2	31.8 ± 16.1	0.279^‡^
	(4–64)	(1–30)	
Number of times driving per month (day), mean ± SD (range)	24.3 ± 30.9	23.8 ± 9.5	0.177^‡^
	(1–30)	(1–30)	
Time spent driving per day (minutes), mean ± SD (range)	110.8 ± 132.3	120.9 ± 127.4	0.252^‡^
	(15–480)	(15–540)	

SD = standard deviation. Data are shown as mean ± standard deviation.

‡Mann–Whitney U test

†Fisher's exact test

The average number of all types of accidents per person was significantly higher in the PH+ tests (4.2±1.7) than the PH- tests (0.3±0.8) (P<0.001, Wilcoxon signed-rank test). For all 8 scenarios, the number of accidents was significantly higher in the PH+ tests than the PH- tests (Table 2).

**Table 2:** Comparison of collisions between PH- and PH+.

	PH- (n = 88)	PH+ (n = 88)	P value
Oncoming right-turning vehicles (2 scenarios)			
A. Oncoming right-turning blue car	5 (5.7%)	32 (36.4%)	<0.001^†^
B. Oncoming right-turning blue car	6 (6.8%)	33 (37.5%)	<0.001^†^
Suddenly appearing hazards from the side (6 scenarios)			
C. White car approaching from the left	5 (5.7%)	74 (84.1%)	<0.001^†^
D. Red car approaching from the left	3 (3.4%)	84 (95.5%)	<0.001^†^
E. Mobility scooter approaching from the left at an unmarked crossing	1 (1.1%)	13 (14.8%)	0.0012^†^
F. Blue car approaching from the left at an unmarked crossing	3 (3.4%)	56 (63.6%)	<0.001^†^
G. Mobility scooter approaching from the right	0 (0%)	17 (19.3%)	<0.001^†^
H. Blue car approaching from the right crossing	1 (1.1%)	61 (69.3%)	<0.001^†^

†Fisher's exact test

In the PH10 group, the average number of all types of accidents per person was significantly higher for the PH+ tests (4.59± 1.81) than the PH- tests (0.24 ±0.73) (P<0.001). In reviewing each scenario, we found that the number of accidents with PH10 was significantly higher in all scenarios than in PH- tests (Table 3).

**Table 3:** Comparison of collisions between PH10 and PH- in 51 subjects and between PH15 and PH- in 37 subjects.

	PH10 group			PH15 group		
	PH- (n = 51)	PH+ (n = 51)	P value	PH- (n = 37)	PH- (n = 37)	P value
Oncoming right-turning vehicles (2 scenarios)						
A. Oncoming right-turning blue car	3 (5.9%)	18 (35.3%)	0.002^†^	2 (5.4%)	14 (37.8%)	0.0013^†^
B. Oncoming right-turning blue car	2 (3.9%)	23 (45.1%)	<0.001^†^	4 (10.8%)	10 (27.0%)	0.1361^†^
Suddenly appearing hazards from the side (6 scenarios)						
C. White car approaching from the left	3 (5.9%)	48 (94.1%)	<0.001^†^	2 (5.4%)	26 (70.3%)	<0.001^†^
D. Red car approaching from the left	2 (3.9%)	50 (98.0%)	<0.001^†^	1 (2.7%)	34 (91.9%)	<0.001^†^
E. Mobility scooter approaching from the left at an unmarked crossing	0 (0%)	11 (21.6%)	<0.001^†^	1 (2.7%)	2 (5.4%)	1.000^†^
F. Blue car approaching from the left at an unmarked crossing	2 (3.9%)	35 (68.6%)	<0.001^†^	1 (2.7%)	21 (56.8%)	<0.001^†^
G. Mobility scooter approaching from the right	0 (0%)	16 (31.4%)	<0.001^†^	0 (0%)	1 (2.7%)	1.000^†^
H. Blue car approaching from the right crossing	0 (0%)	33 (64.7%)	<0.001^†^	1 (2.7%)	28 (75.7%)	<0.001^†^

†Fisher's exact test

In the PH15 group, the average number of all types of accidents per person was significantly higher in the PH+ tests (3.68± 1.49) than PH- tests (0.32 ±0.88) (P<0.001). In reviewing each scenario, we found that the number of accidents in 5 of 8 scenarios was significantly higher in the PH+ tests than the PH- tests (Table 3).

The average numbers of all types of accidents per person was significantly higher in the PH10 group (4.59±1.81) than the PH15 group (3.68±1.49) (P = 0.032). The average number of accidents in the 6 scenarios with the sudden appearance of vehicles was significantly higher in the PH10 group (3.78±1.33) compared to the PH15 group (3.02±1.18) (P = 0.020). Table 4 shows the comparison between the PH15 and PH10 groups for each scenario. The number of accidents due to the 2 scenarios with jump-out events from the left or right at an unmarked crossing were significantly higher in the PH10 than the PH15 group, after correction of multiple comparisons with Bonferroni's method. The number of accidents due to the 2 scenarios with oncoming right-turning vehicles were not significantly higher in the PH10 than the PH15 group.

**Table 4:** Comparison of collisions in each scenario for both the PH10 and PH15 groups.

	PH10 group (n = 51)	PH15 group (n = 37)	P value
Oncoming right-turning vehicles (2 scenarios)			
A. Oncoming right-turning blue car	18 (37.8%)	14 (37.8%)	0.8529^†^
B. Oncoming right-turning blue car	23 (45.1%)	10 (27.0%)	0.1184^†^
Suddenly appearing hazards from the side (6 scenarios)			
C. White car approaching from the left	48 (94.1%)	26 (70.3%)	0.0061^†^
D. Red car approaching from the left	50 (98.0%)	34 (91.9%)	0.3054^†^
E. Mobility scooter approaching from the left at an unmarked crossing	11 (21.6%)	2 (5.4%)	0.0646^†^
F. Blue car approaching from the left at an unmarked crossing	35 (68.6%)	21 (51.6%)	0.2709^†^
G. Mobility scooter approaching from the right	16 (31.4%)	1 (2.7%)	0.0007^†^
H. Blue car approaching from the right crossing	33 (64.7%)	28 (64.7%)	0.3507^†^

†Fisher's exact test

## Discussion

In the current study, VF constriction was created artificially by having subjects with a normal VF wear PH glasses, which allows a controlled comparison between full and limited VF in the same drivers. In addition to VF integrity, many other factors, including motor, sensory and cognitive functions, influence an individual’s driving performance [15]. The main advantage of simulating VF damage in normal subjects is that the effects of confounding factors, other than VF damage, can be corrected for when comparing results obtained for the same subjects with and without artificial VF disturbance. However, PHs with the same aperture can cause different individual levels of VF constriction, and, therefore, we devised PHs with variable apertures. PHs with variable apertures can be made to simulate concentric constriction of binocular VF as measured with a multiple-stimulus self-check VF screener (CLOCK CHART^®^) [17]. Wood JM et al. [20] reported on young persons with normal eyesight wearing PHs, and investigated the importance of the VF on driving performance by measuring the impact of VF constriction on road driving. They found that constriction of the binocular VF to 40° or less significantly increased the time taken to complete a driving course, and reduced the ability to detect and correctly identify road signs, avoid obstacles, and maneuver through narrow areas. However, the study did not evaluate the relationship between traffic accidents and constriction of the binocular VF.

In the current study, we examined the relationship between the number of accidents and 2 types of visual fields, comparing the number of accidents between each constricted VF with the number of accidents with no constricted vision. We simulated concentric constriction of binocular VF to central 15° and 10° in a wide age range population with normal eyesight. We identified relationships between traffic accidents and constriction of the binocular VF from PHs using a DS system. In this study, the number of accidents was significantly higher in the PH+ compared with the PH- group (P<0.001) in all 8 scenarios. Comparing the PH15 and PH10 groups, the average number of all types of accidents per person was significantly higher in the PH10 group than the PH15 group.

The number of accidents due to collisions with oncoming right-turning vehicles was significantly greater in both the PH10 and PH15 groups compared with the PH- group, while no differences were noted between the PH10 and PH15 groups. Kunimatsu-Sanuki et al. [3] investigated the association between visual field defects and collisions with oncoming right-turning cars in patients with advanced glaucoma using a Honda Safety Navi system. They found that the number of accidents due to collisions with oncoming right-turning cars was significantly associated with decreased inferior binocular integrated visual field mean sensitivity within 13 to 24 degrees of the fixation point, older age, and worse visual acuity. Our finding that the number of accidents due to collisions with oncoming right-turning cars was significantly associated with peripheral visual field outside of 15° is consistent with their findings.

As the PH10 and PH15 experiments were not always conducted at the same time of day, caution should be exercised when directly comparing the PH10 and PH15 results [18,19]. However, we found significantly higher accident numbers for 2 scenarios of jump-out events, with a hazard suddenly appearing from the left or right, in the PH10 group than in the PH15 group. One of the 2 scenarios involves a white car approaching from the left and the other is a mobility scooter approaching from the right. Kunimatsu-Sanuki et al. [2] used a Honda Safety Navi system and compared the number of collisions between patients with advanced glaucoma and age-matched and driving exposure time-matched normal subjects. The authors reported that the number of collisions was significantly higher for patients with advanced glaucoma than for normal control subjects (P<0.0001). Moreover, the authors also found the incidence of collisions with a white car approaching from the left was not significantly higher for patients with advanced glaucoma than for normal control subjects (advanced glaucoma: 41.7%, normal control: 22.2%, P = 0.13) [2]. In our study, the number of accidents in the scenario involving a white car approaching from the left was significantly higher in the PH15 and PH10 groups than in subjects without PH glasses. This is possibly due to the VF impairment caused by PH10 or PH15 in our study being more severe than that of advanced glaucoma in the previous study [2]. Glaucoma patients with central visual disturbance do not necessarily exhibit peripheral VF loss [21]. In the study by Kunimatsu-Sanuki et al., the incidence of collisions with a mobility scooter approaching from the right was significantly higher for patients with advanced glaucoma than for normal control subjects (advanced glaucoma: 22.2%, normal control: 0%, P = 0.0051) [2]. In the same scenario, the number of accidents was significantly higher in the PH10 group than the PH- group (PH10: 31.4%, PH-: 0%, P<0.001), consistent with Kunimatsu-Sanuki et al. [2]. However, the number of accidents was similar in the PH15 and PH- groups in the same scenario (PH15: 2.7%, PH-: 0%, P = 1.00). Taken together, the results of the current study suggest that the visual field of 10° to 15° was more important for avoiding collisions in this scenario than peripheral VF loss.

The DS test findings indicate that collisions were significantly more likely with the use of PH glasses in certain types of situations, particularly with vehicles suddenly appearing from the side of the street. In the PH15 group, the scenarios with higher accident rates were those involving driving on a straight road with a good view, but not at a crossing, in a crosswalk, or at a road traffic sign. Restricting the VF to only 10° significantly increased the risk of collisions even further. There are various factors affecting situations that result in traffic accidents. Our results suggest that crossings, crosswalks, and traffic signs alert drivers and prepare them for the possibility of vehicles suddenly appearing from the side of the street.

Our study has several limitations. First, because we used the same course for both the PH+ and PH- testing experiences, a learning effect may have occurred in subjects. However, the influence of the visual field constriction has not been overestimated because we conducted the test with PHs before the test without PHs.

Second, the PH10 and PH15 groups did not include the same subjects. However, there were no significant differences in age, refractive error, gender, driving history, number of times driving per month, and time spent driving per day between the two groups.

Third, driving performance has been shown to be affected by circadian rhythm [18, 22]. We cannot exclude the possibility that our results were influenced by the effect of circadian rhythm on driving performance. However, the influence of circadian rhythm on intra-subject comparisons was likely minimal, as participants were examined with and without PH glasses during the same time frame, and participants reporting fatigue were also excluded. Although DS tests with and without PH glasses were performed in the same subjects within 30 minutes, DS tests with PH10 and PH15 were not always performed at the same time of the day. Thus, caution should be exercised when directly comparing the PH10 and PH15 groups [18,22]. The current study focused mainly on comparing results obtained with and without the use of PH glasses, where effects of circadian rhythm were minimized.

The fourth limitation of this study is that the DS experience does not fully reflect an on-road real driving situation. Driving is a complex activity involving sensory, motor, and cognitive functions [15,18,19]. Therefore, we investigated control traffic- and automobile-related conditions using DS. We attempted to control inter-individual differences in human-related factors by comparing the results obtained in the same subjects with and without artificial VF disturbance. Glen et al. [23,24] developed an eye-tracking and computer set capable of generating an extensional gaze-contingent scotoma simulation (GazeSS) overlaid on film content, and reported that simulated VF loss impaired hazard detection on their system. Our study also showed that concentric constriction of the VF was associated with an increased number of traffic accidents, confirming impaired hazard detection by VF loss.

It should be noted that subjects could notice the VF defects created by PH glasses or GazeSS [23,24], while glaucoma patients generally do not perceive their scotomas [25–27]. By constructing binocular central VFs from bilateral monocular VFs [2,28,29], it may be possible to recruit patients with concentrically constricted integrated binocular VFs. In such cases, however, normal subjects without VF impairment would be needed as a separate control group, and it would therefore become difficult to control for inter-individual differences in human-related factors.

Furthermore, subjects with a restricted VF may attempt to compensate the restricted VF with additional eye and head movements [30,31], which were not considered in the present study. Although we did not monitor the fixation point, as was done in previous studies [22,24,30–34], subjects with PH glasses could only see the screen through the PHs; and only when looking forward. Thus, we believe that the effects of compensatory eye movements were minimal under the conditions of the current experiment.

We simulated concentric constriction of binocular VF with PHs in subjects with normal eyesight in this study, and evaluated the influence of concentric constriction of the VF on driving based on review of the number of accidents during the DS tests. The study results demonstrate the drastic effect of VF loss on driving ability, but the numbers likely represent a worst-case scenario because mitigating factors in real-world driving were not considered. Our DS system did not use the subject’s operation of the steering wheel or the accelerator. The subjects were only required to watch the screen and brake to avoid collisions [2,3]. Although DS systems exhibit advantages in controlling external conditions and in simulating traffic accidents [1,13,14], DS systems cannot duplicate the on-road real driving situation [18, 19]. Regardless, the results of the present study provide useful information that needs to be reconfirmed in a future road test.

## Conclusions

In conclusion, PHs, which can be used to create specific visual angles by adjusting the aperture, were useful in evaluating the effects of peripheral VF on automobile driving. The concentric constriction of VF was associated with an increased number of traffic accidents. Restricting the VF to only 10° significantly increased the risk of collisions even further. The DS findings indicate that concentric constriction of the VF almost inevitably leads to collisions in those types of situations where vehicles suddenly appear from the side of the street. Our study showed that the visual field of 10° to 15° was important to avoid collisions in places with straight roads and good views.

## Abbreviations

VF: visual field
DS: driving simulator
PH: pinhole
MVAs: motor vehicle accidents
HFA 24–2: Humphrey Visual Field Analyzer Swedish Interactive Threshold Algorithm standard 24–2
BCVA: best-corrected visual acuity
